# *In Vitro* Phosphorylation and Acetylation of the Murine Pocket Protein Rb2/p130

**DOI:** 10.1371/journal.pone.0046174

**Published:** 2012-09-24

**Authors:** Muhammad Saeed, Florian Schwarze, Adele Loidl, Joachim Meraner, Markus Lechner, Peter Loidl

**Affiliations:** Division of Molecular Biology, Biocenter, Innsbruck Medical University, Innsbruck, Austria; Peking University Health Science Center, China

## Abstract

The retinoblastoma protein (pRb) and the related proteins Rb2/p130 and 107 represent the “pocket protein” family of cell cycle regulators. A key function of these proteins is the cell cycle dependent modulation of E2F-regulated genes. The biological activity of these proteins is controlled by acetylation and phosphorylation in a cell cycle dependent manner. In this study we attempted to investigate the interdependence of acetylation and phosphorylation of Rb2/p130 *in vitro*. After having identified the acetyltransferase p300 among several acetyltransferases to be associated with Rb2/p130 during S-phase in NIH3T3 cells *in vivo*, we used this enzyme and the CDK4 protein kinase for *in vitro* modification of a variety of full length Rb2/p130 and truncated versions with mutations in the acetylatable lysine residues 1079, 128 and 130. Mutation of these residues results in the complete loss of Rb2/p130 acetylation. Replacement of lysines by arginines strongly inhibits phosphorylation of Rb2/p130 by CDK4; the inhibitory effect of replacement by glutamines is less pronounced. Preacetylation of Rb2/p130 strongly enhances CDK4-catalyzed phosphorylation, whereas deacetylation completely abolishes *in vitro* phosphorylation. In contrast, phosphorylation completely inhibits acetylation of Rb2/p130 by p300. These results suggest a mutual interdependence of modifications in a way that acetylation primes Rb2/p130 for phosphorylation and only dephosphorylated Rb2/p130 can be subject to acetylation. Human papillomavirus 16-E7 protein, which increases acetylation of Rb2/p130 by p300 strongly reduces phosphorylation of this protein by CDK4. This suggests that the balance between phosphorylation and acetylation of Rb2/p130 is essential for its biological function in cell cycle control.

## Introduction

Soon after the discovery of the tumor suppressor retinoblastoma protein (pRb), two other proteins sharing the characteristic structural and functional properties of pRb were identified [1]–[3]; these were termed Rb1/p107 and Rb2/p130; together with the founder protein pRb, these proteins represent the pocket protein family. Highlighted by the fact that one or more pocket proteins are mutated in almost all known cancer types, pocket proteins play an important role in regulating cellular homeostasis. By their ability to modulate expression from E2F-dependent promoter sites and the capability to inhibit CDK2, pocket proteins control crucial events like progression through the cell cycle, growth suppression, differentiation, development, senescence, apoptosis and DNA-repair [4]–[6].

Rb2/p130 is a nuclear phosphoprotein, sharing homology within the pocket domain with both other family members but being more closely related to Rb1/p107 than to pRB. As both other members of the pocket protein family Rb2/p130 is phosphyorylated in a cell cycle dependent manner by cyclin-dependent kinases (CDKs); more than 20 distinct residues have been identified as phosphorylation sites [7]–[11]. The majority of these sites can be phosphorylated by either CDK-2, -4 or 6, while 5 residues are the target of another kinase [10]. It was shown that phosphorylation of p130 by CDKs predisposes the protein for ubiquitination and thus proteosomal degradation [12]. However, in certain cell types phosphorylated Rb2/p130 persists until G_2_-period [13]. In contrast to the common picture of pocket protein inactivation through phosphorylation by CDKs, p130 associates with E2F-4 in a distinct phosphorylation state as cells enter G_0_ [7]; this modification state is independent from CDK activity and has been ascribed to glycogen synthase kinase 3 [14]. Mapping of phosphorylation sites revealed only 3 out of 22 CDK consensus sites being conserved between pRB and p130 whereas 10 phosphorylated serine/threonine residues are conserved between p107 and p130, indicating pronounced differences in the functional consequences of modification among the three pocket proteins.

We have recently discovered that hyperphosphorylated Rb2/p130 exists in an acetylated form in NIH3T3 cells which is exclusively located in the nucleus; acetylation is cell cycle dependent, starting in S-phase and persisting until late G_2_-period [13]. Using recombinant Rb2/p130 and truncated versions for acetylation by the acetyltransferase p300, a total of 5 acetylation sites were identified; predominant acetylation was pinpointed to the C-terminal lysine residue K1079, whereas minor modification occurs on K1068 and K1111 as well as on the N-terminal residues K128 and K130 [13]. Although acetylation was only found in hyperphosphorylated Rb2p130, it remained unknown whether phosphorylation is a prerequisite for acetylation or *vice versa*.

In the present study we addressed the question whether phosphorylation and acetylation of Rb2/p130 are mutually interdependent. By immunoprecipitation we identified the acetyltransferase p300 to be associated with Rb2/p130 *in vivo*. We then analyzed acetylation and phosphorylation by p300 and CDK4 of a variety of recombinant Rb2/p130 proteins with mutations in 3 acetylatable lysine residues and in truncated versions.

## Results

### The Acetyltransferase p300 and the Deacetylase HDAC II are Associated with Rb2/p130 during S-phase in NIH3T3 Cells

In a first step we attempted to identify an acetyltransferase responsible for acetylation of Rb2/p130 *in vivo*, in order to use this enzyme for our *in vitro* modification studies.

**Figure 1:**
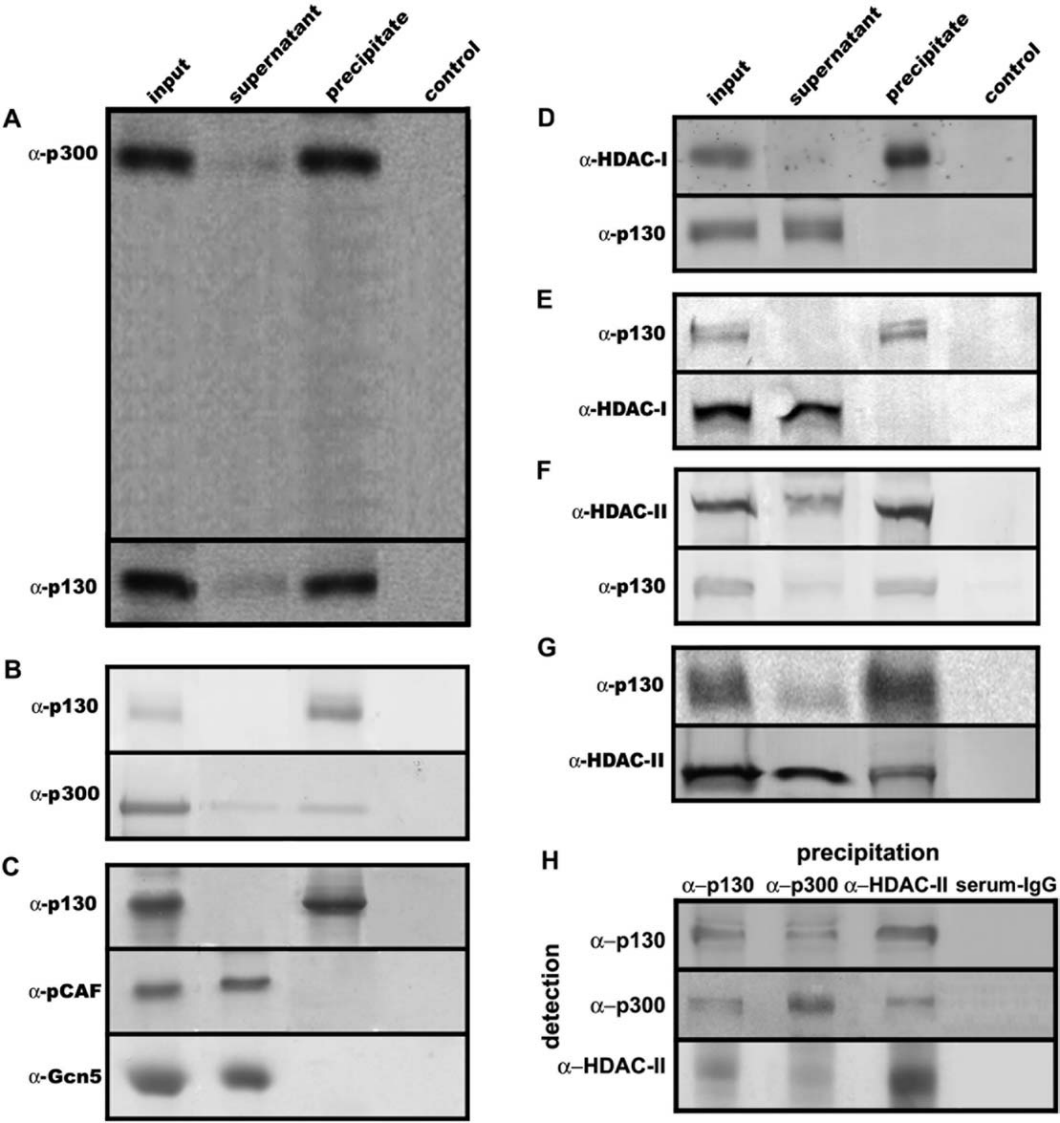
p300 and HDAC II are associated with Rb2/p130 during S-phase in NIH3T3 cells. Nuclear extracts of NIH3T3 cells in S-phase (16 h after re-addition of fully supplemented medium) were immunoprecipitated with antibodies against p300, HDACI, HDAC II, pCAF, Gcn5 and Rb2/p130 respectively, coupled to Protein-G-Sepharose beads. After washing, beads (precipitate), supernatant and nuclear extracts (input) were subjected to SDS-PAGE with subsequent immunoblotting against the indicated antibodies. Protein-G-Sepharose beads without antibodies were used as control. Antibodies used for immunoprecipitation were: anti-p300 (**A**), anti-Rb2/p130 (**B, C, E, G**), anti-HDAC I (D), anti-HDAC II (**F**). (**H**), S-phase nuclear extracts of NIH3T3 cells were subjected to anion exchange chromatography and subsequent size exclusion chromatography. A fraction corresponding to ∼850 kDa was used for precipitation with either anti-p130, anti-p300 or anti-HDAC-II antibodies, respectively and subsequent detection with these antibodies after SDS-PAGE and western blotting. For control rabbit serum total IgG were used instead of primary antibodies for precipitation.

Nuclear extracts of NIH3T3 cells synchronized in S-phase were incubated with antibodies against a variety of histone acetyltransferases or deacetylases. The immuno-precipitates were then analyzed for the presence of Rb2/p130. These experiments only revealed p300 to be associated with Rb2/p130. **Figure 1A** shows that anti-p300 antibodies almost completely co-precipitated Rb2/p130. Likewise, anti-Rb2/p130 antibodies co-precipitated a significant amount of p300 (**Figure 1B**). When anti-Rb2/p130 antibodies were used for precipitation, neither pCAF nor Gcn5 could be detected in the immunoprecipitate (**Figure** 1C). Corresponding immuno-precipitation experiments were performed using antibodies against class I histone deacetylases (HDACs). **Figure 1D** shows that anti-HDAC I antibodies did not co-precipitate Rb2/p130; in line with this, anti-Rb2/p130 antibodies could not co-precipitate HDAC I (**Figure** 1E). Figure **1F** demonstrates that anti-HDAC II antibodies co-precipitated Rb2/p130; likewise anti-Rb2/p130 antibodies co-precipitated HDAC II (**Figure 1G**). These results provide cirumstantial evidence that p300 and HDAC II are associated with Rb2/p130 in S-phase cells and may therefore be responsible for acetylation and deacetylation of Rb2/p130.

To substantiate this finding we extracted nuclear proteins from S-phase cells and purified high molecular weight protein complexes by sequential separation with anion exchange- and size exclusion chromatography. Size exclusion chromatographic fractions containing protein complexes of ∼750 kDa that contained Rb2/p130 were subjected to immunoprecipitation using antibodies against Rb2/p130, p300 and HDAC II, respectively. **Figure** 1H shows that these *in vivo* complexes contain Rb2/p130, p300 and HDAC II. Although this is not a proof that these three proteins are part of the same protein complexes, the result provides a strong indication.

We could not detect p300 and HDAC II in immunoprecipitates when using nuclear extracts of serum-starved, arrested NIH3T3 cells (result not shown). In line with these results, we had already shown that p300 is able to acetylate Rb2/p130 as well as truncated versions of the protein *in vitro* [13].

### Effect of Replacement of Lysines 128, 130 and 1079 by Arginines or Glutamines on *in vitro* Acetylation of Rb2/p130 by the Acetyltransferase p300

**Figure 2:**
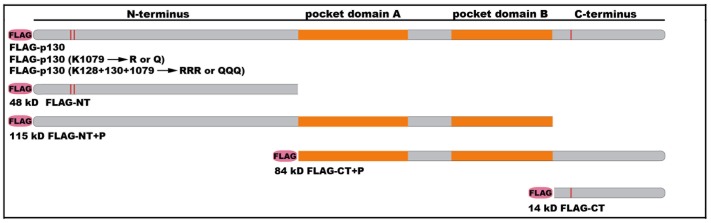
Expression of recombinant FLAG-tagged Rb2/p130 proteins using the baculovirus expression system. Schematic representation of the different full length and truncated versions of Rb2/p130. Mutations of acetylatable lysine residues that were mutated to arginine or glutamine are indicated. The approximate position of lysines 128, 130 and 1079 respectively, are marked by red lines. Molecular size of the truncated proteins is given in kD. C-terminus (CT); N-terminus (NT); pocket domain (P).

To study *in vitro* modification of Rb2/p130 by the acetyltransferase p300 and the protein kinase CDK4/cyclin D1, we expressed different full length and truncated FLAG-tagged Rb2 proteins in insect cells using the baculovirus expression system. **Figure 2** schematically depicts the recombinant proteins: full length Rb2/p130 (FLAG-p130), the N-terminus (FLAG-NT), the N-terminus + pocket domain (FLAG-NT+P), the C-terminus + pocket domain (FLAG-CT+P), and the C-terminus (FLAG-CT). In addition, FLAG-tagged full length versions were expressed where certain C-terminal or/and N-terminal lysines were mutated to either arginine or glutamine. A total of 9 recombinant Rb2 proteins were then used for our *in vitro* modification studies.

**Figure 3:**
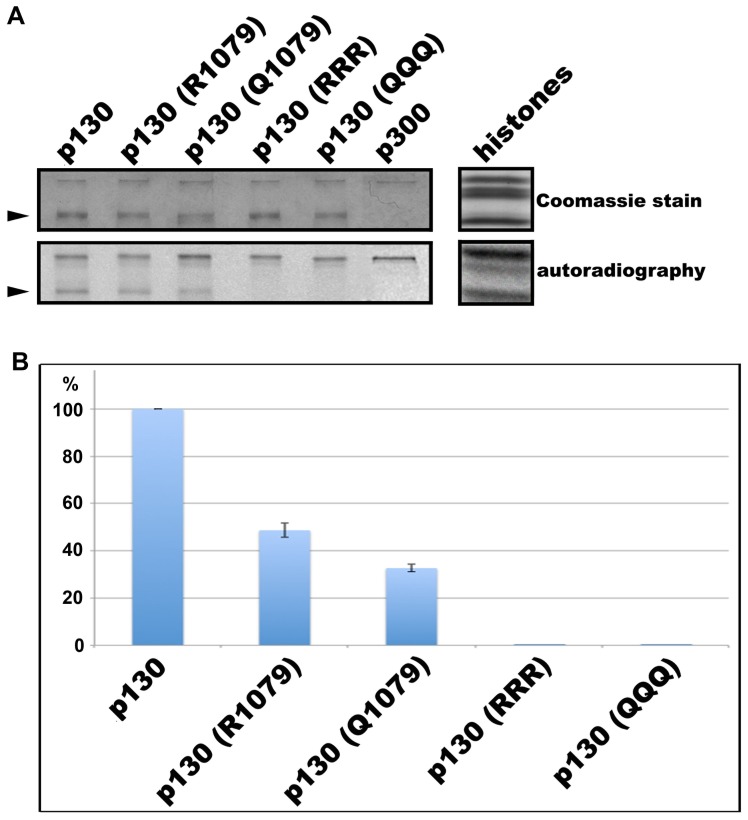
Effect of exchange of lysines 128, 130 and 1079 by arginines or glutamines on *in vitro* acetylation of Rb2/p130 by p300. (A) The different proteins were incubated with p300 and ^14^C-labelled acetylCoA; assay products were subjected to SDS-PAGE with subsequent autoradiography. Arrows indicate the position of Rb2/p130. The slower migrating band is p300. Histones served as a positive incorporation control. (B) Gels/autoradiograms of three independent experiments were analyzed by densitometry using Image-Quant software (Molecular Dynamics) for quantitation. Control (p130) is 100%.

Since we had previously demonstrated that K1079 is the main site of Rb2/p130 acetylation [13], we investigated the effect of a mutation of that particular residue to either a basic, charged amino acid (arginine; equivalent to unmodified lysine with respect to charge) or a non-charged amino acid (glutamine; equivalent to acetylated lysine with respect to charge). Incubation of full length Rb2/p130 with the acetyltransferase p300 and ^14^C-labelled acetylCoA with subsequent autoradiography of SDS-polyacrylamide gels resulted in radioactivity incorporation in Rb2/p130 as already reported [13]. **Figure 3A** shows that mutation of lysine 1079 to arginine as well as to glutamine significantly reduced the level of acetylation, indicating that the residual acetylation in the mutant proteins is due to modification of the N-terminal lysine residues 128 and 130. Mutation of neither lysine 1068 nor 1111 affected the level of acetylation (results not shown); accordingly, mutation of lysines 128, 130 and 1079 to arginine (p130 RRR) or glutamine (p130 QQQ) completely abolished acetylation (**Figure 3A**). To monitor the efficiancy of p300 we included core histones as substrate; moreover, auto-acetylation of p300 can be clearly seen in all lanes (**Figure 3A**; upper band in each panel). Densitometric analysis of 3 independent experiments using Image-Quant software showed that mutation of lysine 1079 to arginine reduced acetylation to 49% of the control, whereas mutation to glutamine caused a reduction to 33% (**Figure** 3B).

### *In vitro* Phosphorylation of Rb2/p130, Truncated and Mutated Versions by CDK4

**Figure 4:**
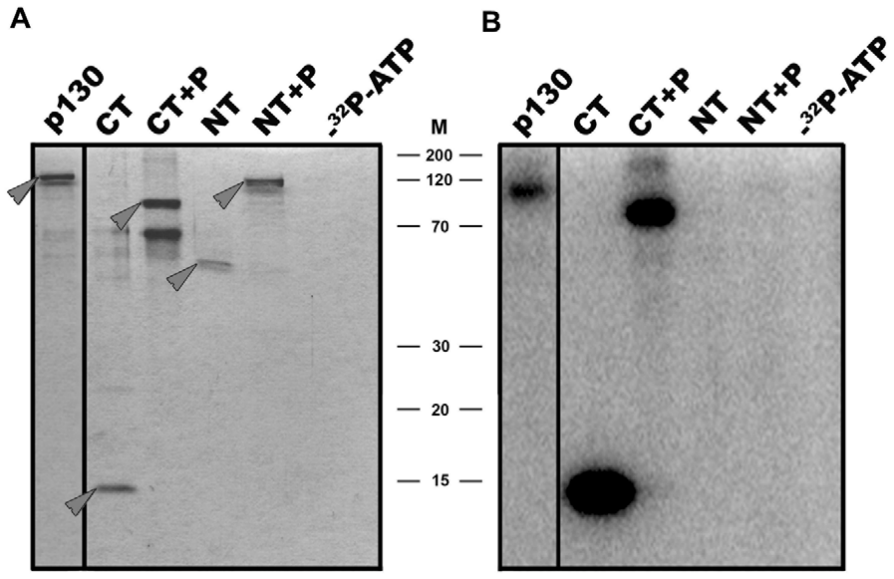
*In vitro* phosphorylation of full length Rb2/p130 and truncated versions by CDK4. Recombinant full length Rb2/p130, the C-terminus (CT), the C-terminus + pocket domain (CT+P), the N-terminus (NT) and the N-terminus + pocket domain (NT+P) were subjected to CDK4 protein kinase assays using ^32^P-labelled ATP. Samples were then subjected to SDS-PAGE with subsequent autoradiography. (**A**), Coomassie-Blue stained gel; (**B**), autoradiogram. Arrows in (**A**) mark the position of the recombinant Rb2/p130 proteins. Assay sample without ^32^P-labelled ATP served as negative control. M, positions of marker proteins indicated.

We then investigated phosphorylation of our recombinant Rb2 proteins by CDK4. Incubation of full length Rb2/p130 with CDK4/cyclinD1 and ^32^P-labelled ATP resulted in strong phosphorylation (**Figure 4**). The truncated C-terminal versions of the protein (C-terminus alone, C-terminus + pocket domain) were even phosphorylated to a higher extent as compared to full length Rb2/p130, whereas the truncated N-terminal forms (N-terminus alone, N-terminus + pocket domain) were not phosphorylated (**Figure 4**).

**Figure 5:**
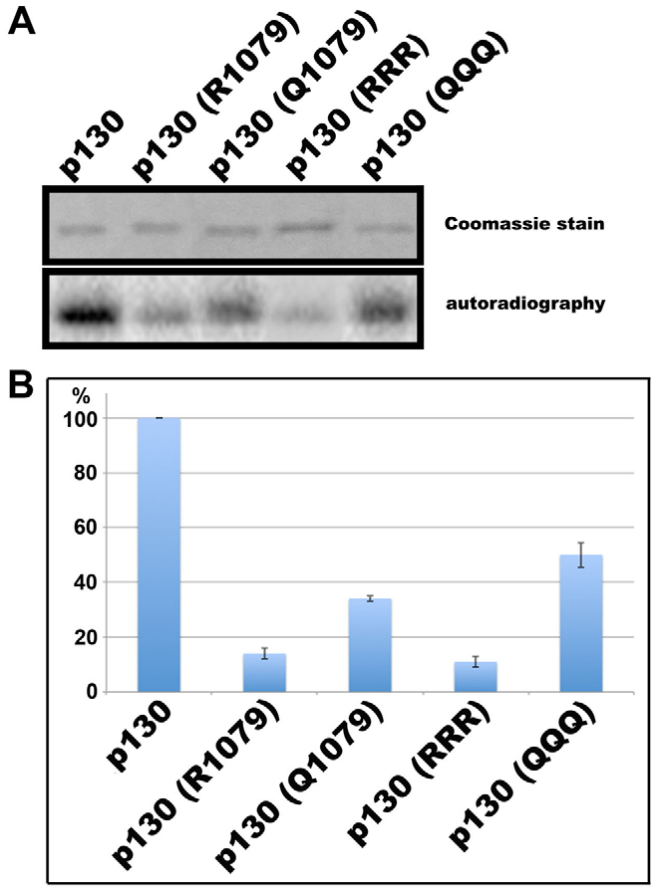
*In vitro* phosphorylation of full length Rb2/p130 and mutated versions by CDK4. Recombinant full length Rb2/p130 and mutated proteins with exchange of lysine 1079 to arginine or glutamine (p130-R1079, p130-Q1079) and with exchange of three lysines (1079, 128, 130) to arginine or glutamine (p130-RRR, p130-QQQ) were used in CDK4 protein kinase assays with ^32^P-labelled ATP. Samples were then subjected to SDS-PAGE with subsequent autoradiography. Coomassie-Blue stained gel (upper panels); autoradiogram (lower panels). (**B**) Gels/autoradiograms of three independent experiments were analyzed by densitometry using Image-Quant software (Molecular Dynamics) for quantitation. Control (p130) is 100%.

In order to analyze whether mutations in the acetylatable lysine residues affect phosphorylation of Rb2/p130, we performed CDK4 protein kinase assays with normal and mutated full length Rb2/p130 proteins. **Figure 5A** shows that mutations in lysine 1079 significantly reduced phosphorylation by CDK4; interestingly, exchange of lysine by arginine caused a more pronounced reduction of phosphorylation as compared to the exchange by glutamine. Mutation of all three lysines (128, 130 and 1079) had a similar effect; again replacement by arginine had a stronger effect as compared to glutamine (**Figure 5A**). Densitometric analysis of 3 independent experiments using Image-Quant software showed that mutation of lysine 1079 to arginine reduced phosphorylation to 14% of the control, whereas mutation to glutamine caused a reduction to 34% (**Figure** 5B). Replacement of all three lysines by glutamine reduced phosphorylation to 50%, whereas replacement by arginine resulted in reduction to 11% (**Figure 5B**).

### Human Papilloma Virus 16-E7 Protein Reduces *in vitro* Phosphorylation of Rb2/p130

**Figure 6:**
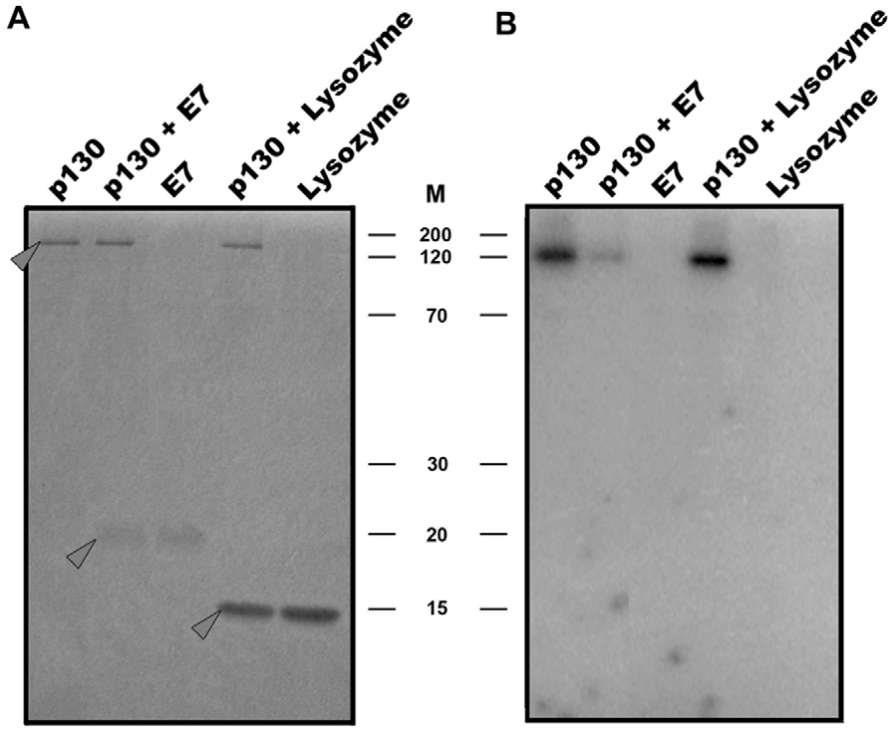
Human papilloma virus 16-E7 protein reduces *in vitro* phosphorylation of Rb2/p130. Recombinant full length Rb2/p130 was used in CDK4 protein kinase assays in the presence and absence of HPV16-E7 and lysozyme, respectively. Samples were then subjected to SDS-PAGE with subsequent autoradiography. (**A**), Coomassie-Blue stained gel; (**B**), autoradiogram. Arrows mark the position of Rb2/p130, HPV16-E7 and lysozyme, respectively. M, positions of marker proteins indicated.

Pocket proteins have been shown to be targets of small DNA virus oncoproteins, such as proteins from certain types of papilloma-, polyoma- and adeno-viruses [15], [16]. Human papilloma virus 16-E7 protein (HPV 16-E7) binds to p130 and usually targets it for degradation; moreover, histone deacetylases bind to p130 and the E7 protein, thereby inhibiting histone deacetylase binding to the E2F2 promoter and elevating the acetylation of histones associated with E2F-responsive promoters [17]. We recently showed that HPV 16-E7 enhances the acetylation of Rb2/p130 by the acetyltransferase p300 *in vitro* [13]. To analyze whether HPV 16-E7 has any effect on Rb2/p130 phosphorylation *in vitro*, we did CDK4 protein kinase assays in the presence of the E7 protein. **Figure 6** shows that E7 drastically reduced phosphorylation of Rb2/p130 which is contrary to its effect on acetylation. As a control we used lysozyme as a comparable small molecular weight protein; lysozyme had no effect on Rb2/p130 phosphorylation by CDK4 *in vitro*.

### Mutual Interdependence of Acetylation and Phosphorylation of Rb2/p130

**Figure 7:**
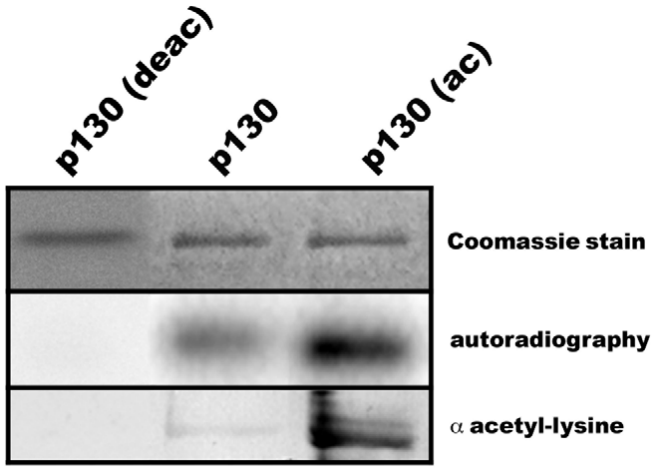
Acetylated Rb2/p130 is strongly phosphorylated by CDK4. Recombinant full length Rb2/p130 was either acetylated *in vitro* by p300 and non-radiolabelled acetyl-CoA (p130-ac) or deacetylated *in vitro* by HDAC2 (p130 (deac). The efficiancy of the acetylation/deacetylation reaction was checked by immunoblotting with anti-acetyllysine antibodies. Subsequently, control, acetylated and deacetylated Rb2/p130 were used in CDK4 protein kinase assays with ^32^P-labelled ATP. Assay products were analyzed by SDS-PAGE (Coomassie Blue panel) with subsequent autoradiography.

Since mutation of acetylatable lysines to arginine or glutamine had such a pronounced effect on CDK4-catalyzed Rb2/p130 phosphorylation *in vitro*, we set out to study a possible interdependence of both modifications in more detail. To this end we acetylated recombinant full length Rb2/p130 by p300 and non-radioactive acetyl-CoA and then used the acetylated protein as substrate in CDK4 protein kinase assays (**Figure 7**). The acetylation extent was checked by immunoblotting using anti-acetyllysine antibodies. The recombinant baculovirus protein had a low background acetylation whereas the *in vitro* acetylated protein yielded a prominent immunosignal with the anti-acetyllysine antibody. It is clearly evident that acetylation of Rb2/p130 strongly enhances its phosphorylation by CDK4 *in vitro*. To further substantiate this finding we subjected recombinant Rb2/p130 to deacetylation by purified HDAC 2 of NIH3T3 cells before using it as substrate in the CDK4 protein kinase assay. Deacetylation of Rb2/p130 completely abolished subsequent phosphorylation (**Figure 7**).

**Figure 8:**
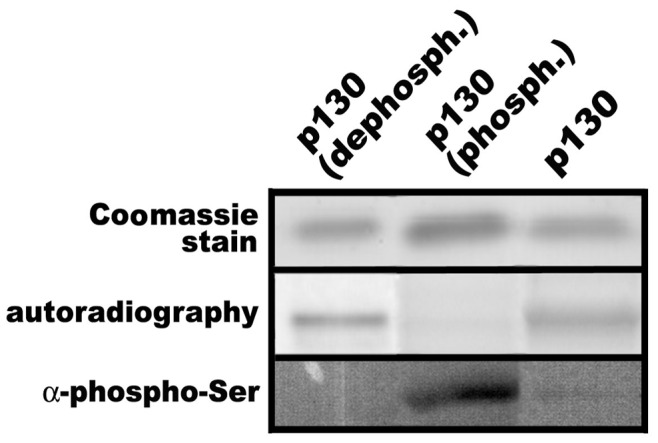
Phosphorylation of Rb2/p130 abolishes *in vitro* acetylation by p300. Recombinant full length Rb2/p130 was either dephosphorylated by alkaline phosphatase (p130-dephosph.) or subjected to CDK4 protein kinase assays with non-radioactive ATP (p130-phosph.). Phosphorylation was determined by anti-phospho-serine immunoblotting. Control p130, phosphorylated p130 and dephosphorylated p130 were used as substrates in p300 acetyltransferase assays with ^14^C-labelled acetyl-CoA. Samples were then analyzed by SDS-PAGE with subsequent autoradiography.

To analyze the effect of the phosphorylation status on Rb2/p130 acetylation by p300 we used three Rb2/p130 protein substrates in the acetyltransferase assay. i) Recombinant full length Rb2/p130, ii) Rb2/p130 that was dephosphorylated *in vitro* by alkaline phosphatase, iii) Rb2/p130 that was phosphorylated *in vitro* by CDK4. The phosphorylation status was checked by immunoblotting with anti-phospho-serine antibodies. **Figure 8** shows that recombinant baculovirus expressed Rb2/p130 has a significant background level of phosphorylation; alkaline phosphatase completely erased phosphorylation (p130-dephosph.), whereas incubation with CDK4 and non-radioactive ATP resulted in efficient phosphorylation. These 3 proteins, differing in their phosphorylation status, were then used as substrates in the acetyltransferase assay with p300 and ^14^C-acetylCoA. **Figure 8** shows that dephosphorylation of Rb2/p130 slightly enhanced its acetylation, whereas phosphorylation by CDK4 abolished *in vitro* acetylation.

## Discussion

We have recently shown that Rb2/p130 is posttranslationally acetylated in a cell cycle dependent manner [13]; acetylation was restricted to hyperphosphorylated p130 forms and the acetylated p130 was exclusively located in the nucleus despite the fact that the major part of hyperphosphorylated p130 forms resided in the cytoplasm. Although both the hyperphosphorylated as well as the acetylated forms of p130 appeared in G1/S and stayed on an almost constant level until into G_2_-period, it remained elusive, whether phosphorylation and acetylation occur independent from each other or are mutually affected by each other. The results presented in this paper rather suggest an interdependence of both modifications; the fact that deacetylated Rb2/p130 is not phosphorylated by CDK4, whereas increasing levels of acetylation are paralleled by increasing phosphorylation argues for acetylation to be a prerequisite for phosphorylation. This assumption is corroborated by the finding that only dephosphorylated Rb2/p130 is acetylated by p300, while phosphorylation by CDK4 abolishes the acetylation potential. We therefore speculate that Rb2/p130 acetylation precedes phosphorylation *in vivo*, although additional, yet unknown mechanisms could be necessary for *in vivo* phosphorylation.

Circumstantial support for acetylation being a prerequisite for phosphorylation also comes from the results presented in **Figure 5**. When the major acetylatable lysine 1079 is exchanged by arginine (which represents the unmodified form of lysine with respect to charge in the lysine side chain), we observed a strong inhibition of phosphorylation by CDK4; on the other hand exchange by glutamine (which rather mimicks the acetylated state of lysine with respect to charge neutralization in the lysine side chain) resulted in a much less pronounced inhibition of phosphorylation. This might indicate that a charge change at that particular amino acid position in the C-terminus is a prerequisite for phosphorylation. It seems possible that lysine 1079 acetylation induces a conformational change that facilitates CDK 4 binding and enzymatic action. It was already pointed out that the C-terminus of Rb2/p130 is a particularly important regulatory region that contains several crucial posttranslationally modifed amino acids [13]: acetylated lysine 1079, phosphorylated serines 1064, 1076 and 1108 [10], and neighboring proline residues 1065, 1077 and 1109; the proline residues might be substrates of peptidyl-proline isomerases, which would induce potential conformational changes. It should be mentioned in this context that the C-terminus of other important regulatory proteins acting in transcriptional control and cell cycle regulation contains multiple amino acid residues that are subject to posttranslational modifications by otherwise chromatin modifying enzymes, *e. g.* p53 [18].

Moreover, proline 1077 and lysine 1079 are located in one of two nuclear localization sequences, flanked by phosphorylated serine 1064. Transfection experiments with p130/GFP constructs where lysine 1079 was mutated to arginine or glutamine did not change the nuclear localization of the reporter protein (Schwarze and Loidl, unpublished), which is in line with functional deletion studies demonstrating that deletion of one of the two C-terminal nuclear localization sequences was not sufficient to change nuclear localization of Rb2/p130 [19]. Recently, it was shown that the serine/threonine phosphatase PP2A can stabilize Rb2/p130 by dephosphorylation at serine 1080 and threonine 1097 in human ovarian carcinoma cells [20] by mediating nuclear import of the protein; acetylation of a lysine residue within this nuclear localization sequence might have a role in this stabilization process. The fact that we find HDAC II to be associated with Rb2/p130 may indicate that the acetylation state of lysine 1079 has to be dynamically balanced in order to exert a regulatory function. It is equally possible that binding of HDAC II to Rb2/p130 simply reflects a targeting function so that Rb2/p130 leads HDAC II to certain gene targets; it is well known that pRB family members bind class I HDACs [17].

For Rb2/p130 it has been shown that phosphorylation by CDKs predisposes the protein for ubiquitination and subsequent proteosomal degradation [12]. The fact that hyperphosphorylated, acetylated Rb2/p130 persists until into G_2_ period in the nuclei of NIH3T3 cells [13] might be interpreted as a stabilizing effect of acetylation on phosphorylated Rb2/p130, thus protecting a certain population of Rb2/p130 molecules from proteasomal degradation. It is worth mentioning in this context, that expression of a mutated form of Rb2/p130 under a conditional promoter in NIH3T3 cells (lysine1079 mutated to arginine) severely perturbed cell cycle progression, as investigated by FACS analysis; expression of Rb2/p130 mutated at position 1068 (lysine replaced by arginine) did not have an effect on cell cycle progression (Saeed and Loidl, unpublished), supporting a functional role of lysine 1079 acetylation *in vivo*.

Based on the data presented in this paper and previously published results [13], we assume that the coordinated acetylation and phosphorylation of Rb2/p130 is important for its role in cell cycle regulation. The fact that HPV 16-E7 inhibits phosphorylation of Rb2/p130 although it enhances acetylation is in line with this assumption; HPV 16-E7 leads to heavily acetylated p130 molecules that are not hyperphosphorylated, thereby creating a modification status that does not represent the physiological condition. The unphysiological modification status of Rb2/p130 caused by the E7 protein thus contributes to cell cycle deregulation by human papilloma virus.

## Materials and Methods

### Cell Culture

NIH3T3 cells (German Collection of Microorganisms and Cell Cultures, No. ACC59) were grown in DMEM/HAMs 15-K medium supplemented with 10% (v/v) fetal calf serum and 1 mM L-glutamine. Synchronization was achieved by serum starvation for 72 h; synchronous growth was induced by re-addition of fully supplemented medium.

For expression of recombinant proteins, Sf9 cells (from *Spodoptera frugiperda*) were cultivated in Gracès insect cell medium at 27°C and ambient atmosphere.

Cell lines were routinely tested for contamination with mycoplasms using commercially available kits.

### Recombinant Protein Expression

Sf9 cells susceptible to infection with *Autographa california* multiple nuclear polyhedrosis virus (AcMNPV baculovirus) were transfected using Cellfectin Transfection Reagent (Invitrogen, No 10362-010). Cells were seeded at a density of 1.5×10^6^ per 35 mm dish 1 h before transfection. Cellfectin and the bacmid-minipreparation (pFAST-Bac 1, Invitrogen) were diluted in Grace medium and complex formation was allowed for 45 min before addition of 800 µl of medium and cells; cells were incubated for 5 h at 27°C in the transfection medium, after which cells were shifted to normal growth medium for 72 to 96 h. Several rounds of virus amplification were performed until recombinant protein levels were sufficient.

### Cellular Fractionation and Protein Extraction

For preparation of nuclear extract fractions of NIH3T3 cells a commercially available kit was used (NE-PER^TM^Nuclear and Cytoplasmic Extraction Reagents, Pierce, No. 78733).

For extraction and purification of recombinant proteins from Sf9 cells, the cell pellets were resuspended in lysis buffer (10 mMTris-HCl, pH 8.7, 500 mM NaCl, 10% (v/v) glycerol, 0.1% (v/v) Nonidet NP-40, 10 mM 2-mercaptoethanol, 2 mM PMSF, 1×protease inhibitor cocktail (Roche, No 1873580); 1 ml lysis buffer per 140 mm round dish). Cells were lysed in a pre-cooled 15 ml douncer. The lysate was centrifuged twice at 1000×*g*, the supernatant transferred into a fresh tube in-between. 150 µl (300 µl 50% slurry) of the affinity matrix (FLAG-M2 agarose, Sigma) equilibrated 3 times in 1 ml lysis buffer was added to the supernatant. Binding was performed on an overhead shaker at 25 rpm at 4°C for 3 h. Beads were collected by centrifugation and washed 3 times with lysis buffer (800×*g* for 5 min at 4°C). Purified proteins were either used directly bound to beads or eluted 3 times from the affinity matrix by the addition of FLAG-peptide (Sigma; 0.4 mg/ml FLAG-peptide in lysis buffer) at 4°C for 10 min while gently agitating. Eluted proteins were separated from the beads by centrifugation at 1000×*g* for 5 min.

### Chromatographic Procedures

Nuclear extracts were loaded onto a 1 ml MonoQ HR 5/5 anion exchange column (Amersham Pharmacia Biotech). After washing the column with chromatography buffer (10 mM Tris-HCl, 10 mM NaCl, 0,5 mM EDTA, 10% (v/v) glycerol, pH 7.8) bound proteins were eluted with a linear gradient from 10 to 700 mM NaCl in chromatography buffer. Fractions were assayed for p130. Fractions around 300 mM NaCl containing high amounts of p130 were pooled and applied to a TosoH TSKG4000PWXL size exclusion chromatography column (TosoH Biosep), equilibrated and eluted with chromatography buffer containing 150 mM NaCl. Fractions corresponding to molecular masses of 700 to 800 kDa containing high amounts of p130 were used for immunoprecipitation experiments.

### Immunoprecipitation

Nuclear extract or chromatographic fractions (200 µl) were incubated with antibody solution (2.5 µg/ml) for 60 min at 4°C. Antibodies used were: anti-CBP/p300 (Abcam No ab3164), anti-Rb2/p130 (BD Transduction Laboratories, No 610262; Santa Cruz, No sc-317), anti-HDAC I (Zymed-Invitrogen, No 34–8300), anti-HDAC II (Zymed-Invitrogen, No 34–6400), anti-pCAF (Abcam No ab12188) and anti-Gcn5 (Abcam No ab18381). Protein G Sepharose (GE-Healthcare Amersham; 30 µl) equilibrated in IP-buffer (phosphate buffered saline, 1% (v/v) Triton-X-100, 1 mM dithiothreitol, 0.2 mM phenylmethylsulfonylfluoride, 1 mM NaF) was added and incubated for 2 h at 4°C under gentle shaking, followed by centrifugation at 800×*g* for 5 min. The supernatant was saved for SDS-PAGE and immunoblotting. Beads were washed 4 times with 0.5 ml IP-buffer and finally resuspended in 50 µl of SDS-sample buffer for further SDS-PAGE and immunoblotting. Control precipitations were performed with nuclear extracts and Protein G Sepharose without antibodies.

### *In vitro* Protein Modification Assays

Acetylation of recombinant Rb2 proteins (control or mutated versions) was performed by incubation of recombinant proteins bound to FLAG agarose beads in buffer P (50 mM Tris, pH 8.0, 150 mM NaCl, 5% glycerol, 0.1 mM Trichostatin A, 10 mM EDTA, 10 mM dithiothreitol) with 20 ng of p300 and 20 µM ^14^C-acetylCoA for 30 min at 37°C (final volume 50 µl). For pre-acetylation with subsequent phosphorylation radioactive acetylCoA was replaced by non-radioactive acetylCoA. The acetylation reaction was stopped by chilling on ice and either addition of SDS-sample buffer (for further SDS-PAGE and autoradiography) or by washing the beads 3 times with an excess of buffer K for subsequent phosphorylation reactions.

Phosphorylation of recombinant Rb2 proteins (full length, truncated or mutated versions) was performed by mixing 200 ng of recombinant protein (3 µl) with 10 µl of buffer K (30 mM Tris-HCl, pH 8.0; 2 mM MgCl_2_, 30 mM NaCl, 0.2 mM dithiothreitol, 1 mM EGTA), 50 µM non-radiolabelled ATP, 20 ng CDK4/CycD1 (Proquinase GmbH, No 10142-0143-1) and 10 µCi of gamma^32^P-labelled ATP. For pre-phosphorylation with subsequent acetylation radioactive gamma^32^P-labelled ATP was omitted. The assay mixture was incubated for 30 min at 37°C. The reaction was terminated by chilling on ice and either addition of SDS-sample buffer (for further SDS PAGE and autoradiography) or by washing the beads 3 times with an excess of buffer P for subsequent acetylation reaction.

For certain experiments recombinant Rb2/p130 bound to FLAG agarose beads were subjected to dephosphorylation by alkaline phosphatase or to deacetylation by HDAC2. For dephosphorylation 1 µl of alkaline phosphatase was used (Fermentas, No. EF0341) at 37°C for 45 min. After incubation 5 mM EDTA was added and the reaction mixture was heated to 70°C for 5 min. Then the mixture was washed 3 times with an excess of ice cold buffer P and used for subsequent acetylation reactions. For deacetylation purified HDAC2 from NIH3T3 cells was used at 30°C for 30 min. After incubation the reaction mixture was chilled on ice and washed 3 times with an excess of ice cold buffer P before used for subsequent phosphorylation reactions.

### Electrophoresis, Immunoblotting and Autoradiography

Samples were subjected to SDS-10%-polyacrylamide gel electrophoresis (SDS-PAGE). Gels were stained with Coomassie Blue. For immunoblotting proteins were transfered to nitrocellulose membrane. Visualization of antibody binding was done with alkaline phosphatase conjugated secondary antibodies or electrochemi-luminescence. Autoradiography was performed by exposing gels on phospho-imaging plates that were analysed in a STORM phosphoimager system (Molecular Dynamics). Stained gels and blots were analyzed in a laser densitometer (Molecular Dynamics). Quantitative evaluation was done with Image-Quant software (Molecular Dynamics).
